# Socioeconomic deprivation and dementia risk in New Zealand: A modelling study

**DOI:** 10.1177/00048674261470616

**Published:** 2026-08-02

**Authors:** Etuini Ma’u, Nicholas Rush, Jade Tamatea, Malakai ‘Ofanoa, Sebastian Walsh, Gill Livingston, Naaheed Mukadam, Gary Cheung, Sarah Cullum

**Affiliations:** 1 Department of Psychological Medicine, University of Auckland, New Zealand; 2 Te Whatu Ora Waikato, Hamilton, New Zealand; 3 Te Kupenga Hauora Māori, University of Auckland, New Zealand; 4 School of Population Health, University of Auckland, New Zealand; 5 Cambridge Public Health, University of Cambridge, UK; 6 Division of Psychiatry, University College London, UK; 7 North London NHS Foundation Trust, St Pancras Hospital, London, UK; 8 Te Whatu Ora Te Toka Tumai Auckland, New Zealand; 9 Global Brain Health Institute (GBHI), Trinity College Dublin, Ireland

**Keywords:** dementia, risk factors, prevention, ethnicity, New Zealand, Maori, Pacific, equity, socioeconomic deprivation, population attributable fraction

## Abstract

**Introduction:**

We aimed to estimate the population attributable fraction for dementia associated with neighbourhood deprivation and the potential impact of reducing deprivation on dementia prevalence over time.

**Methods:**

We calculated the population attributable fraction across Aotearoa New Zealand for 13 risks (less education, hypertension, obesity, smoking, depression, physical inactivity, social isolation, diabetes, excessive alcohol, air pollution, hearing loss, vision loss, high low-density lipoprotein cholesterol) stratified by deprivation and ethnicity. We modelled the impact of reducing deprivation on dementia prevalence over 30 years.

**Results:**

Population attributable fraction increased with deprivation, from 36.0% in the least deprived to 45.8% in the most deprived quintile. Modelled reductions in deprivation were predicted to produce greater dementia prevalence reductions at all time points for Māori and Pacific peoples compared to Europeans.

**Discussion:**

Dementia prevention potential increases as deprivation increases. The impact of reducing deprivation increases over 30 years. Projected benefits are disproportionately greater in populations in areas of higher deprivation.

## Introduction

The World Health Organization (WHO, 2017) declared dementia a global health priority in 2017 because of population ageing and the projected huge impact of increasing numbers of people living with dementia. In Aotearoa New Zealand (NZ), the number of people with dementia is projected to double from 83,000 in 2025 to almost 170,000 people in 2050 (Ma’u, 2021b). Primary prevention and risk reduction have the potential to delay or prevent the development of dementia in many individuals, reducing population prevalence projections, and the subsequent impact on families, health and social services (Long et al., 2023).

The 2024 Lancet commission on dementia report identified 14 potentially modifiable risk factors for dementia with sufficient evidence to support a causal relationship and estimated that 45% of dementia worldwide is theoretically avoidable if all were eliminated (Livingston et al., 2024). In NZ, we demonstrated ethnic differences in dementia risk, with the dementia prevention potential higher in Māori (51.4%) and Pacific peoples (50.8%) than in NZ European (47.6%) and Asian peoples (40.8%), because of a higher prevalence of potentially modifiable risk factors (Ma’u et al., 2021a). Ethnic differences in dementia risk factors have also been demonstrated in other countries such as Australia (Thompson et al., 2022), Brazil (Suemoto et al., 2023) and the United States (Lee et al., 2022). We have modelled that a partial (25%) reduction in midlife dementia risk could meaningfully reduce dementia prevalence in NZ, with almost 12,000 fewer cases at 30 years, and benefits were greatest in Māori and Pacific peoples (Ma’u et al., 2025b).

Individuals who are more socioeconomically deprived are at greater risk of dementia (Bodryzlova et al., 2023; Wang et al., 2023). In NZ specifically, recent studies have reported that those in the most socioeconomically deprived decile had a 50% higher risk of dementia compared to those in the least deprived decile (Ma’u et al., 2024a). Socioeconomic deprivation in NZ has also been shown to be associated with both lower baseline cognitive function and more rapid cognitive decline (Rohr et al., 2024). Within NZ, Māori and Pacific peoples experience substantially more socioeconomic deprivation compared to Europeans (Loring et al., 2022), contributed to by structural differences such as the effects of migration (Lotoala et al., 2014) and colonisation (Reid et al., 2019) and racism (Harris et al., 2019). Furthermore, most dementia risk factors are more common in people with higher socioeconomic deprivation. These lead to Māori and Pacific peoples experiencing poorer health outcomes than Europeans, reflected in a shorter life expectancy and a disproportionate burden of illness (Ministry of Health (MoH), 2018).

The aim of this study was to explore the association between socioeconomic deprivation and dementia in the NZ population and within the four main NZ ethnic groups- Māori, Pacific, European and Asian people. We aimed to calculate the population attributable fraction (PAF) for dementia at different levels of deprivation; to estimate the risk of dementia associated with different levels of deprivation and model the impact of reducing deprivation in midlife on dementia prevalence projections.

## Methods

### Ethics approval

This study received ethics approval from the University of Auckland (REF: AH2974 & AH23608) and Statistics New Zealand (REF: MAA2022-03).

### Calculating the PAF at different levels of deprivation

#### Risk factors for dementia

We followed the risk factor definitions in our previous NZ study (Ma’u et al., 2021a). Briefly, we used prevalence data from the annual New Zealand Health Survey (NZHS), a methodologically rigorous and nationally representative MoH data collection tool used to monitor population health and inform health policy by collecting information including population health, health risk and protective factors, and health service usage (Reach Aotearoa (n.d.)). The target population is the NZ ‘usually resident population’ of all ages, with approximately 99% of the population eligible. The NZHS response rate has consistently been 79% to 80% since the 2011/2012 survey (Reach Aotearoa (n.d.)). A multi-stage, stratified, probability-proportional-to-size (PPS) sampling method is designed to yield an annual sample size of approximately 14,000 participants aged 15 years and older, approximately 0.4% of the usually resident population, where everyone has the same probability of being selected. To ensure adequate estimates, Pacific peoples and Asian ethnic groups were oversampled by increasing probabilities of selection in areas where they are more populous. For Māori, oversampling used the electoral roll rather than an area-based approach. The electoral roll is used to select a sample of addresses where a person has self-identified as having Māori ancestry (MoH, 2011, 2016).

The NZHS includes data on 13 of the 14 dementia risk factors identified in the Lancet Commission on dementia 2024 report, disaggregated by age, sex and deprivation but does not have traumatic brain injury (Livingston et al., 2024). The risk factor definitions we used in our study, consistent with previous papers (Ma’u et al., 2021a, 2025) are presented in Table 1.

**Table 1.** Definitions of risk factors for dementia.

Risk factor	Definition
Less education	Self-reported highest education attained is less than upper secondary (age < 15 years)
Hypertension	Systolic blood pressure ⩾ 140 mm Hg
Obesity	Body mass index (BMI) ⩾ 30 Kg/m^2^
Smoking	Identifies as a current smoker
Depression	Self-reports of ever being diagnosed with depression by a doctor
Physical inactivity	Does not meet WHO physical activity guidelines for exercise of > 30 min a day for at least 5 days a week
Social isolation	Lives alone
Diabetes	Self-reports of ever being diagnosed with diabetes by a doctor
Excessive alcohol	Consumes > 17 units of alcohol a week (1 unit = 10 g pure alcohol)
Air pollution	Urban residence (living in an area with > 10,000 population)
Hearing loss	Self-reports difficulty hearing, even when using a hearing aid
Vision loss	Self-reports difficulty seeing, even if wearing glasses
High low-density lipoprotein cholesterol	Self-reports of ever being told by a doctor that they have high blood cholesterol levels

#### Risk factor relative risk and communality

We used the risk factor relative risks as the Lancet Commission on dementia report 2024 (Livingston et al., 2024) (See Supplementary Table S1). To account for an individual having multiple risk factors that often overlap and cluster, we calculated the communality of risk factors, as described in the Lancet Reports (Livingston et al., 2024) and then weighted PAF. Communality calculates the extent of the shared variance between risk factors and allows for the calculation of an adjusted PAF, weighted by each factor’s unique risk contribution. We used the mean of the 13 communality calculations as the estimated communality for TBI, in line with previous analyses (Livingston et al., 2020), as there is no data set containing all 14 risk factors. These calculations were used as the communality for all subgroup analyses by ethnic groups and level of deprivation.

##### Ethnicity

To ensure comparability across studies, we used the same approach for ethnicity as previously described (Ma’u et al., 2021a). NZ is a multicultural country, and individuals can identify with more than one ethnic group. For analysis, we used the MoH approach of prioritised ethnicity to ensure independence between groups. This means individuals are allocated to only one of the groups they identify with in the prioritised order of Māori, Pacific peoples, Asian and European (MoH, 2017).

#### Neighbourhood deprivation

The 2018 NZ Deprivation Index (NZDep2018) is an area measure of relative socioeconomic deprivation in NZ and provides small geographical area indexes of socioeconomic deprivation and is available in the NZHS. NZDep2018 geographical areas generally contain between 100 and 200 people and are based on “meshblocks,” geographic areas defined by Statistics New Zealand, varying in size from small part of a city to large areas of rural land (Salmond and Crampton, 2012). Using New Zealand census data, the NZDep2018 includes variables intended to be direct measures of deprivation, and are related to income, employment, communication, support, qualifications, owned home, living space, and living conditions of individuals living in each area (Supplementary Appendix Table S2) (Atkinson et al., 2020).

Each meshblock receives a deprivation score based on the weighted average of the included variables, which are then ranked. For our study, we have used NZDep quintiles, which categorise the population into five relative deprivation groups from quintile 1 (least deprived) to quintile 5 (most deprived).

#### Statistical analysis

We calculated an unadjusted and adjusted PAF using the method described in the Lancet commission report, briefly through calculating relative risk and prevalence of each risk factor, then adjusting for communality (see Supplementary Appendix) (Livingston et al., 2020).

### Calculating hazard ratios for dementia at different levels of deprivation

Baseline characteristics, dementia case ascertainment and longitudinal follow-up of the cohort have been previously described (Ma’u et al., 2024a). Briefly, the population at risk is defined as all participants aged >30 years in the NZHS 2011/2012–2018/2019 without a pre-existing diagnosis of dementia. Cases of incident dementia were identified from the Statistics New Zealand (Stats NZ) Integrated Data Infrastructure (IDI), which is a large database holding de-identified data about people and households in NZ (Statistics New Zealand (n.d.)) Each person in the IDI is allocated a Unique Person Identifier. This allows linkage of the NZHS, seven health data sets and sociodemographic details. Characteristics of the data sets and identification of dementia diagnoses in each of the data sets have been previously detailed (see Supplementary Appendix https://bmjopen.bmj.com/content/bmjopen/12/9/e062304.full.pdf?with-ds=yes) (Cheung et al., 2022).

Diagnoses were based on ICD-9 or ICD-10AM dementia codes for the Mortality, Privately Funded Hospital Discharges, Programme for the Integration of Mental Health Data (PRIMHD) and Publicly Funded Hospital Discharges, National Needs Assessment and Service Coordination Information System (SOCRATES) data sets. The Pharmaceutical collection contains information on all publicly funded pharmaceuticals dispensed in the community (Statistics New Zealand). Two antidementia medications (donepezil tablets and rivastigmine transdermal patches) are funded in NZ and they can only be prescribed for a diagnosis of dementia. The International Residential Assessment Instrument (interRAI) is a comprehensive, evidence-based, and standardised assessment that is required to access publicly funded long-term community care or aged residential care in NZ (Schluter et al., 2016). The *interRAI* assessment routinely records a diagnosis of “Alzheimer’s disease” or “Dementia other than Alzheimer’s disease.” Any diagnosis of dementia has the date when the diagnosis was entered. Censoring occurred at the earliest occurrence of death, date of first diagnosis of dementia in any of the seven data sets, or end of follow-up (31 March 2022).

In order to ascertain incident cases only, people with a coded diagnosis of dementia in any of the seven data sets dated during or prior to the survey year were excluded at baseline.

#### Statistical analysis

Stata 16 was used for statistical analysis (StataCorp, 2019).

Cox regression models were performed for neighbourhood deprivation quintiles (defined in objective 1), with time to first diagnosis of dementia as the outcome and deprivation as the main independent variable of interest. Model 1 was adjusted for age and sex. Model 2, the full model, was adjusted for age, sex, and all 13 risk factors (defined in objective 1).

### Modelling the impact of a hypothetical intervention reducing deprivation in midlife on dementia prevalence projections

We developed a Markov model using TreeAge Pro (2021) to estimate the projected proportion of dementia that theoretically could be prevented following a hypothetical intervention in midlife (age 40–64 years) that reduced deprivation (Figure S1 in Supplementary Appendix). Base data required for the model and data availability by dementia stage, age band, and ethnicity are described in Supplementary Table S3. The incidence of dementia, annual transition rates between dementia stages (Supplementary Table S4), and excess mortality due to dementia, by dementia stage, were from published literature (Mukadam et al., 2020; Norton et al., 2014). We used NZ data, disaggregated by age band and ethnicity, for annual mortality rates (Ministry of Health (n.d.)), deprivation and population age structure (Satistics New Zealand, 2018).

We converted calculated hazard ratios for neighbourhood deprivation to relative risk, and these were then transformed into risk in the exposed (R_exp_) and unexposed (R_unexp_) (see Supplementary Appendix).

Two models were run:

1. Model 1: The baseline scenario assumes no change in the distribution of neighbourhood deprivation.
2. Model 2: Is where neighbourhood deprivation is reduced in the population aged 40–64 years. We modelled change in dementia prevalence projection following hypothetical midlife interventions that shift the dementia risk associated with deprivation using four different scenarios (Table 2).

**Table 2.** Alternate model scenarios for the change in neighbourhood deprivation.

A	Reducing the risk of the 20% of the population living in quintile 5 to that of those living in quintiles 1–4
B	Reducing the risk of the 40% of the population living in quintiles 4–5 to that of those living in quintiles 1–3
C	Reducing the risk of the 60% of the population living in quintiles 3–5 to that of those living in quintiles 1–2
D	Reducing the risk of the 80% of the population living in quintiles 2–5 to that of those living in quintile 1

Quintiles: 1 (least deprived) to 5 (most deprived).

Both models are run for 30 years, and the difference in modelled prevalence between models 1 and 2 is therefore the proportional impact of the reduction in neighbourhood deprivation on dementia prevalence. A 30-year time horizon was chosen as a reasonable period for the midlife cohort’s remaining life expectancy, and the reduction in deprivation was modelled. Uncertainty in modelled estimates also increases when projecting further into the future, as epidemiological assumptions around parameters such as dementia incidence and progression, as well as mortality, are likely to vary across cohorts. For example, while dementia incidence has been consistently demonstrated to be declining decade on decade over 25 years from 1988 to 2015 (Wolters et al., 2020), there is emerging evidence that this trend may be reversing (Chen et al., 2023).

## Results

### PAF stratified by deprivation

Table 3 presents the prevalence and adjusted PAF (PAF_adj_) for each risk factor, stratified by deprivation quintile. Risk factor prevalence and PAF_adj_ increased with increasing deprivation for all risk factors except for alcohol, which decreased. Compared to quintile 1 (the least deprived), the PAF_adj_ values were several times higher in quintile 5 for some risk factors such as smoking (3.9×) and diabetes (2.7×). The combined PAF_adj_ for all 13 risk factors was 9.8% higher in quintile 5 (45.8%) compared to quintile 1 (36.0%).

**Table 3.** Risk factor prevalence (Prev – %) and adjusted population attributable fraction (PAF_adj_) for the total NZ population, by deprivation quintile.

	Deprivation quintile									
	Q1		Q2		Q3		Q4		Q5	
	Prev	PAF_adj_	Prev	PAF_adj_	Prev	PAF_adj_	Prev	PAF_adj_	Prev	PAF_adj_
Education	19.4%	3.2%	24.1%	3.8%	25.1%	3.9%	34.4%	5.0%	45.1%	6.0%
Hypertension	27.2%	4.3%	34.0%	5.1%	33.3%	4.9%	36.1%	5.2%	35.3%	5.0%
Obesity	29.9%	4.7%	31.8%	4.8%	36.1%	5.3%	41.0%	5.7%	48.2%	6.4%
Smoking	5.0%	0.9%	9.1%	1.6%	9.8%	1.7%	15.3%	2.4%	23.6%	3.5%
Depression	15.1%	3.7%	15.2%	3.6%	18.5%	4.2%	22.4%	4.9%	22.0%	4.7%
Physical inactivity	46.8%	4.8%	48.6%	4.9%	51.7%	5.1%	55.6%	5.3%	61.3%	5.6%
Low social contact	24.8%	4.0%	33.1%	5.0%	36.1%	5.3%	42.6%	5.9%	44.7%	6.0%
Diabetes	5.8%	0.9%	7.8%	1.1%	9.1%	1.3%	14.4%	1.9%	17.6%	2.3%
Air pollution	72.9%	2.1%	72.8%	2.0%	73.6%	2.0%	63.9%	1.7%	75.9%	2.0%
Alcohol	11.4%	0.7%	9.4%	0.6%	10.2%	0.6%	8.9%	0.5%	9.6%	0.5%
Hearing loss	21.5%	2.8%	22.4%	2.6%	24.2%	2.8%	22.0%	2.5%	24.5%	2.7%
Vision loss	16.7%	2.8%	20.6%	3.0%	23.3%	3.3%	26.4%	3.6%	30.3%	4.0%
High cholesterol	35.9%	3.5%	36.0%	3.1%	35.1%	3.0%	38.2%	3.2%	38.4%	3.1%
Total combined PAF_adj_		36.0%		36.0%		38.2%		42.3%		45.8%

Deprivation quintile: Q1 (least deprived) to Q5 (most deprived).

Figure 1 and Supplementary Table S5 present the total adjusted PAF for each ethnic group, stratified by deprivation quintile. All ethnic groups demonstrated an increasing PAF with increasing deprivation.

**Figure 1.**
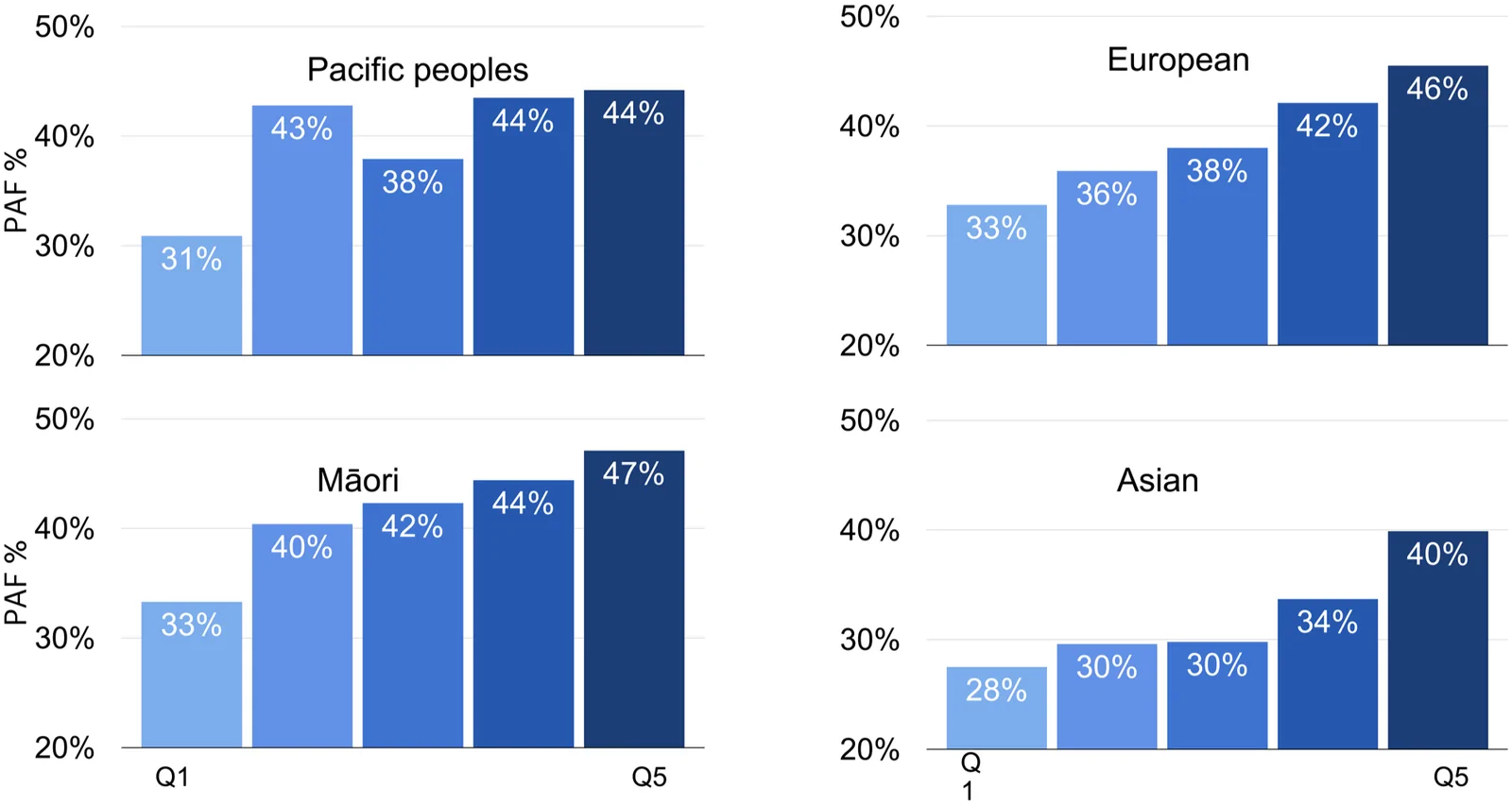
Total population attributable fraction (PAF) for 13 risk factors, by deprivation quintile (Q1 = least deprived, Q5 = most deprived) and ethnicity.

### Hazard ratio of dementia stratified by deprivation

Table 4 reports the hazard ratio of dementia after adjusting for age, sex (model 1) and the 13 risk factors (model 2). Compared to quintile 1 (least deprived), the hazard ratio increased with increasing deprivation from a 1.35 (95% CI 1.15–1.58) times higher risk in quintile 3 to a 1.53 (95% CI 1.31–1.79) times higher risk in quintile 5. The association between risk and deprivation remained after adjusting for the 13 risk factors (model 2). Supplementary Table S6 presents the hazard ratios for the progressive change in deprivation levels.

**Table 4.** Hazard ratio of dementia, adjusting for age, sex, and 13 risk factors, by deprivation quintile.

Deprivation quintile	Model 1		Model 2	
	HR	95% CI	HR	95% CI
Q1 (REF)	1.00		1.00	
Q2	1.18	0.99–1.39	1.13	0.93–1.35
Q3	1.35	1.15–1.58	1.34	1.12–1.58
Q4	1.38	1.18–1.61	1.31	1.10–1.55
Q5	1.53	1.31–1.79	1.45	1.22–1.72

Deprivation quintile: Q1 (least deprived) to Q5 (most deprived). Model 1: adjusted for age and sex. Model 2: adjusted for age, sex, and all 13 risk factors.

### Modelled prevalence projections

Figure 2 presents the modelled change in dementia prevalence projection under four progressively more equalising scenarios. Dementia prevalence reductions occurred in all scenarios, with increasing impact over time. Dementia prevalence reduction in the most progressive scenario was five times higher compared to the least progressive scenario at all time points. Similar patterns are seen when stratifying by ethnicity (Figure 3 and Supplementary Table S7), with the magnitude of prevalence reductions greater for Māori and Pacific peoples compared to Asian and Europeans in all scenarios and at all time points.

**Figure 2.**
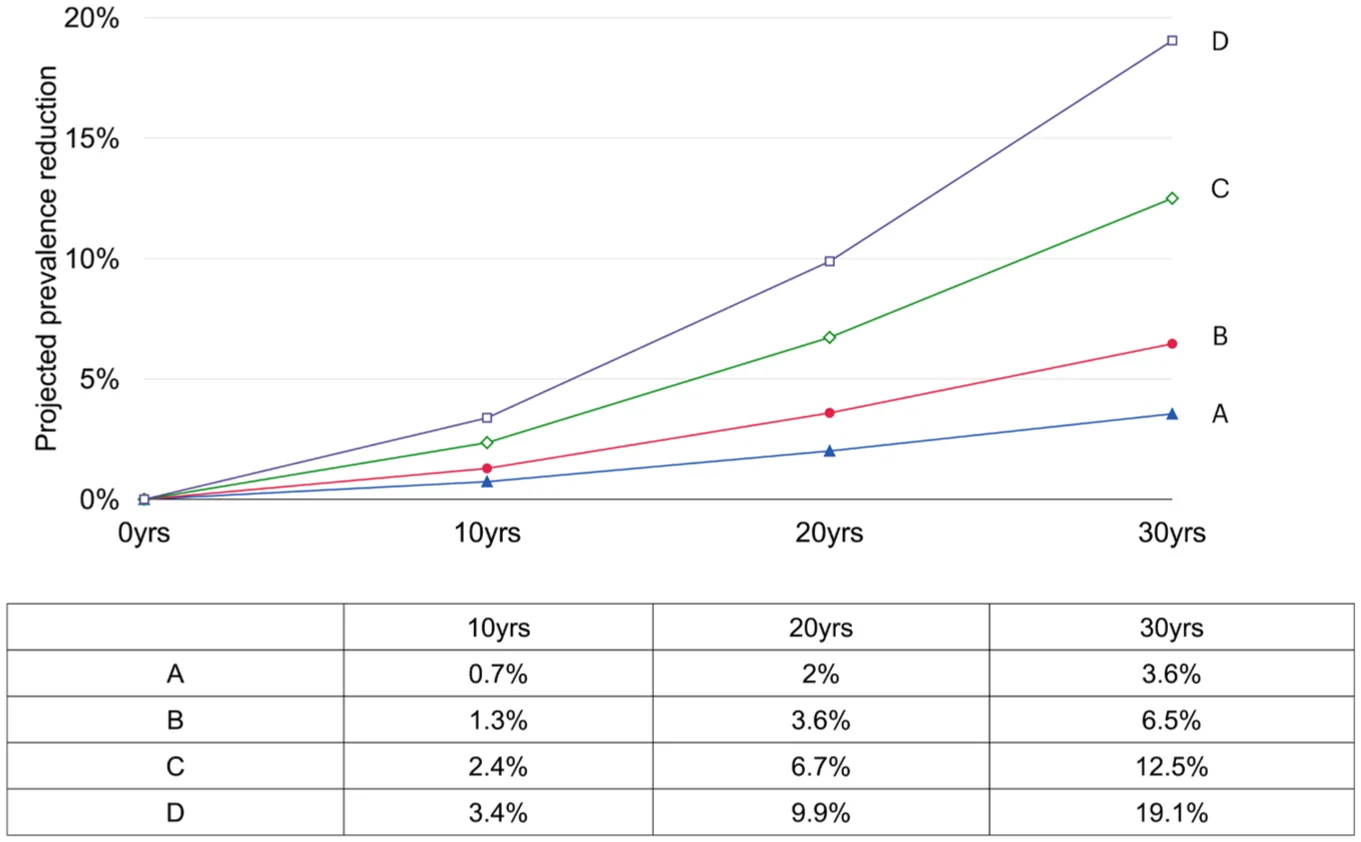
Modelled change in dementia prevalence projection following hypothetical midlife interventions reducing dementia risk associated with deprivation under different scenarios. A if the risk of the 20% most deprived people was reduced to that of the other 80%. B if the risk of the 40% most deprived people was reduced to that of the other 60%. C if the risk of the 60% most deprived people was reduced to that of the other 40%. D if the risk of the 80% most deprived people was reduced to that of the other 20%.

**Figure 3.**
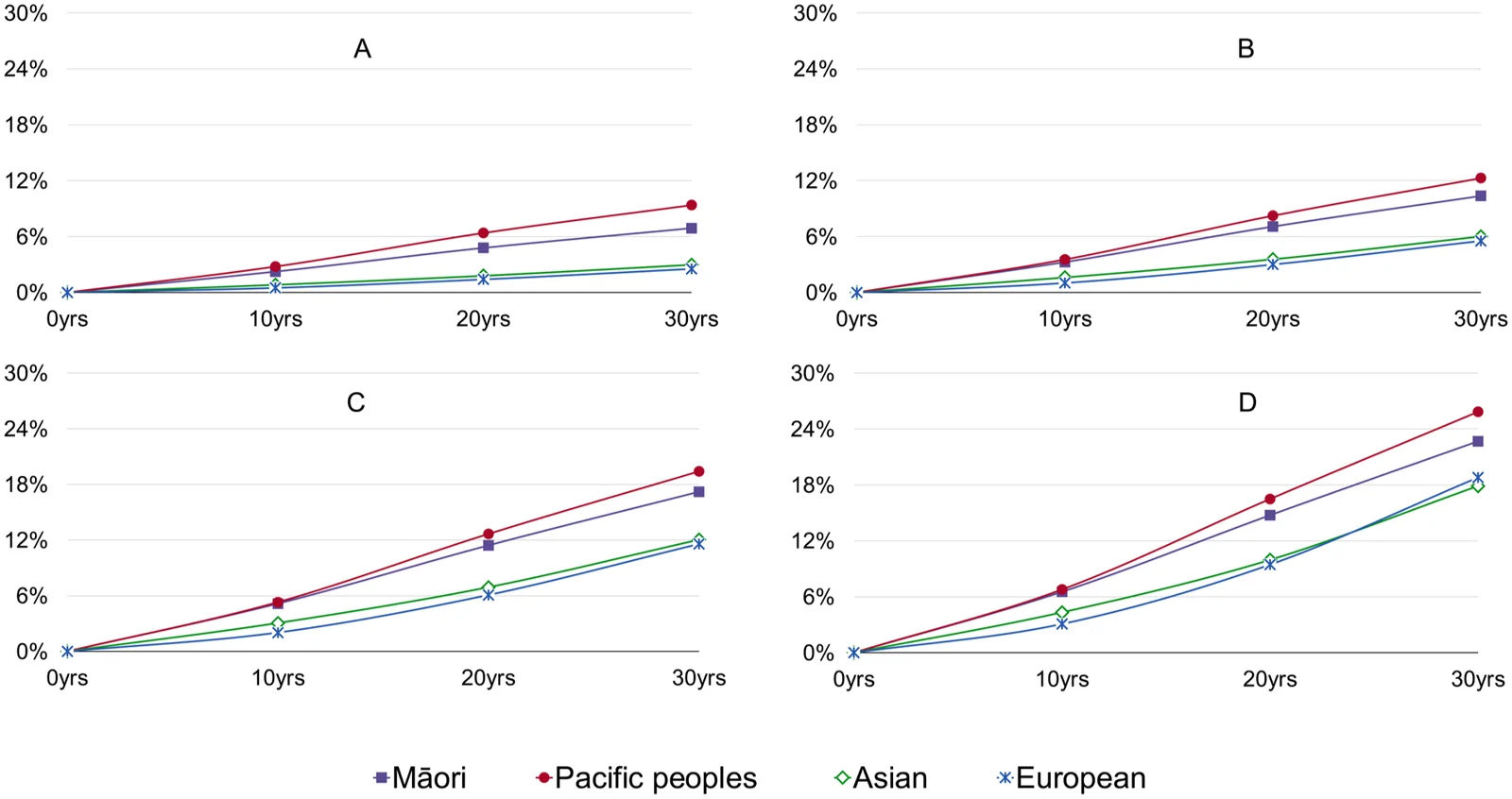
Modelled change in dementia prevalence projections, stratified by ethnicity, following hypothetical midlife interventions that shifts the dementia risk associated with deprivation under different scenarios. A if the risk of the 20% most deprived people was reduced to that of the other 80%. B if the risk of the 40% most deprived people was reduced to that of the other 60%. C if the risk of the 60% most deprived people was reduced to that of the other 40%. D if the risk of the 80% most deprived people was reduced to that of the other 20%.

## Discussion

We found a linear increase in risk factor prevalence with increasing deprivation, with an absolute increase in the combined PAF for 13 risk factors in the total population from 36.0% in quintile 1 (least deprived) to 45.8% in quintile 5 (most deprived). A shift in dementia risk across the population to that of the least deprived 20% of neighbourhoods was associated with significant dementia prevalence reductions at all time points (3.6% at 10 years, 9.9% at 20 years and 19.1% at 30 years). In all scenarios and at all time points, as much as three times greater prevalence reductions were demonstrated for Māori and Pacific peoples compared to Europeans. The total combined PAF at each level of deprivation was similar across the ethnic groups. This indicates that the higher PAF in Māori and Pacific peoples identified previously (Ma’u et al., 2021a) is unlikely to be an innate risk per se rather than explained by an overrepresentation of these groups in areas of higher deprivation, which have higher risk factors for dementia (Loring et al., 2022).

The association of socioeconomic deprivation with dementia risk in this study has also been demonstrated internationally, with similar findings to this study. Using UK biobank data, Klee et al. (2023) found that people living in areas of high socioeconomic deprivation had a 1.47 times higher hazard of dementia compared to those in low-moderate deprivation. The study also demonstrated an increasing dementia hazard with increasing individual-level socioeconomic deprivation independent of area-level deprivation. Dintica et al. (2023) demonstrated an association between an area deprivation index and incident dementia in a US cohort, with a 1.17 times the risk in those living in the highest compared to the lowest area of deprivation, even after adjusting for cardiovascular risk factors. Similarly, differences in the PAFs associated with potentially modifiable risk factors for dementia have been shown to vary by levels of income or deprivation. Studies out of Brazil (Suemoto et al., 2023), Argentina (Calandri et al., 2024), China (Chen et al., 2024) and the United States (Stulberg et al., 2025) all describe income-associated variations in risk factor prevalence and subsequent PAF estimates.

Socioeconomic deprivation is about not only the individual but also the neighbourhood within which they live and work (Klee et al., 2023; Walsh et al., 2022b). Neighbourhood deprivation is associated with higher exposure to obesogenic environments (Giskes et al., 2011), fewer green spaces (Zhang et al., 2024) and higher air pollution (Hajat et al., 2015, Wickham et al., 2023). Our finding of higher risk factor prevalence and PAF with increasing levels of deprivation is in line with local (Stanley et al., 2018) and international (Jivraj et al., 2020) evidence, with all risk factors (except higher alcohol consumption) more prevalent with increasing area deprivation (Broer et al., 2019; Hajat et al., 2015; Hiscock et al., 2012; Kimenai et al., 2022; Long et al., 2023; Selak et al., 2020; Wilson et al., 2006). However, the amount that individual lifestyle factors explain the increased dementia risk associated with deprivation may be small, with a recent UK study estimating that six lifestyle factors mediated < 20% of the risk (Ou et al., 2024; Wang et al., 2024). This is because the increased risk associated with deprivation is not solely due to direct effects on known risk factor prevalence but also indirect effects on risk factor profiles including a longer duration and increased severity of disease (Conrad et al., 2024), timely access to care (Schultz et al., 2018), suboptimal treatment (Delgadillo et al., 2016), and a higher prevalence of disease complications and multimorbidity (Gurney et al., 2020).

We have demonstrated higher prevention potential and projected prevalence reductions for Māori and Pacific peoples compared to Asian and European populations due to both their younger population age structure and greater proportion of people living in areas of high deprivation. While our study was not sufficiently powered to explore the hazard of dementia by both ethnicity and deprivation, it is likely that there is an intersection between these factors. The effects of institutional and structural racism on healthcare access and quality in NZ are well documented (Harris et al., 2018, 2024). Harris et al. (2006) have described the combined effect of deprivation and racial discrimination on inequalities in health outcomes between Māori and NZ Europeans. They showed that while individually adjusting for racial discrimination *or* deprivation reduced the magnitude of inequality, adjusting for both factors *removed* the inequality in health outcomes.

### Strengths and weaknesses

#### NZHS survey methodology

The NZHS is a methodologically rigorous and nationally representative survey which is the best source for prevalence estimates for many health and wellbeing measures in NZ. The data set allows prevalence estimates stratified by prioritised ethnicity and deprivation ensuring fully independent groups for analysis.

Linking the NZHS to seven large, administrative data sets in the IDI allows all participants to be prospectively followed up for incident dementia in multiple data sources. Limitations centre around under reporting of risk factors and of dementia., as well as the absence of primary care data in the IDI (Cheung et al., 2021; Ryan et al., 2022). It has been estimated that these data sets may be missing up to half of prevalent cases (Ma’u et al., 2024b) and a third of incident dementia cases (Ma’u et al., 2024a). There is evidence that the proportion of missing prevalent cases in Non-European ethnic groups are higher due to lower engagement with health services (Ma’u et al., 2024b). The overrepresentation of Māori and Pacific peoples in areas of higher deprivation, in conjunction with the impact of deprivation itself on health service access and utilisation (Schultz et al., 2018), suggests a higher proportion of missing cases in areas of higher deprivation. Lower rates of case ascertainment in those living in areas of higher deprivation may mean the calculated hazard ratios are an underestimate of dementia risk.

The NZHS likely underestimates the prevalence of some risk factors, such as depression, where there are known ethnic disparities in self-reported diagnosis of depression by a doctor relative to screening measures scores for depression (Lee et al., 2017), meaning that prevalence estimates in Non-European ethnic groups are likely to be underestimated. Others, like diabetes and high cholesterol, are self-reported and rely on memory and understanding of the diagnosis and access to appropriate healthcare. We have used proxy measures of cohabitation for social isolation and urban living for air pollution, as there were no direct measures. Assuming those who live alone are socially isolated is not necessarily correct, although there is no consensus on a definition of social isolation, and there is evidence that living alone is associated with increased dementia risk (Desai et al., 2020). The correlation between urban living and air pollution suggests this is a reasonable proxy for air pollution (Chen et al., 2017).

There are also limitations associated with our assumptions about risk factor prevalence and subsequent interventions in midlife. Our PAF estimates and current modelling approach cannot account for the fact that many risk factors are initiated in early life and have an effect throughout the life course (EClipSE Collaborative Members et al., 2010; Muniz-Terrera et al., 2016). Some risk factor interventions, such as educational attainment (Letenneur et al., 1999; Ott et al., 1995), would apply to younger age groups rather than those in midlife, so their impact is unlikely to be evident within our modelled time horizon.

#### Deprivation and ethnicity

There is no single measure that completely captures the socioeconomic position of the NZ population (Jivraj et al., 2020). The indices of NZDep, a small area measure, have excellent construct and criterion validity and compares well with other measures of socioeconomic position such as the NZ Indices of Multiple Deprivation (IMD) in their association with health and social outcomes (Crampton et al., 2019) but more deprived individuals can live in relatively non-deprived areas and vice versa (Salmond and Crampton, 2012). The NZDep is a cross-sectional measure of deprivation and is unable to take into consideration social mobility. As Māori and Pacific peoples are less likely to be upwardly mobile (Morrison et al., 2010; Robertson et al., 2021), the modelled effects of dementia prevention potential associated with reducing deprivation may be underestimates for Māori and Pacific peoples. While we used the standard measure of prioritising ethnicity, thus preventing overlap, people may be of mixed ethnicity.

#### Modelling estimates

We were underpowered to calculate hazard ratios for deprivation stratified by ethnicity so have applied the same risk estimates in our model across all ethnic groups. While we have used incidence rates by 5-year age band for the NZ population aged 60+, this is not available by ethnicity and it is likely these incidence rates are an underestimate for Māori and Pacific peoples (Cheung et al., 2021; Ryan et al., 2022). We were not able to account for the possible impact of reducing deprivation on longevity or mortality rates in those with or without dementia in this model.

### Implications for policy

The interplay between deprivation and dementia is complex and multifactorial, with some arguing deprivation should be considered an explicit and modifiable risk factor in and of itself (Daly, 2024). The evidence for both direct and indirect effects of deprivation on dementia risk highlights the need to use societal approaches to dementia prevention that address the social determinants of health (Walsh et al., 2022a). An emphasis on individual-level lifestyle change is unlikely to be effective without addressing deprivation as a key upstream driver, and mediator, of the social determinants of risk (Rohr et al., 2022).

The disproportionate impact of deprivation on Māori and Pacific peoples highlights the importance of addressing the deeper structural influences on health and wellbeing throughout the life course and their influence on lifetime risk profiles. Interventions targeting healthcare access must not only address the barriers associated with deprivation but also the underlying institutional and structural racism pervading the wider health system (Adkins-Jackson et al., 2023; Curtis et al., 2023).

While our modelling of reduction in deprivation is theoretical, our findings are clear that addressing deprivation would be expected to reduce dementia risk over and above any reduction in risk factor prevalence, and it is likely that the indirect effects of any interventions on general health and wellbeing will provide further benefits.

## Conclusion

This study demonstrates the clear association between socioeconomic deprivation and dementia risk in NZ. This risk is inequitably distributed, disproportionately impacting Māori and Pacific peoples who are overrepresented in areas of higher deprivation. This study also demonstrates that significant reductions in dementia prevalence are possible if deprivation is addressed. This will require a multipronged approach that simultaneously facilitates individual behaviour change while providing an environment that promotes healthy living. Truly addressing socioeconomic deprivation, however, requires us to confront the deeper systemic structural factors perpetuating health inequities. Interventions that successfully lower the risk associated with deprivation could have profound impacts on future dementia prevalence and health equity in NZ.

## Supplemental Material

sj-docx-1-anp-10.1177_00048674261470616 – Supplemental material for Socioeconomic deprivation and dementia risk in New Zealand: A modelling study Supplemental material, sj-docx-1-anp-10.1177_00048674261470616 for Socioeconomic deprivation and dementia risk in New Zealand: A modelling study by Etuini Ma’u, Nicholas Rush, Jade Tamatea, Malakai ‘Ofanoa, Sebastian Walsh, Gill Livingston, Naaheed Mukadam, Gary Cheung and Sarah Cullum in Australian & New Zealand Journal of Psychiatry
