# Supplementary material for: Socioeconomic deprivation and dementia risk in New Zealand: A modelling study
Source: Aust N Z J Psychiatry. 2026 Aug 2;60(11):1063–73. doi: 10.1177/00048674261470616 (PMC13716974; doi:10.1177/00048674261470616)
Supplement: Supplementary file 1 [file sj-docx-1-anp-10.1177_00048674261470616.docx]

**Supplementary Appendix**

**Table s1: Risk factor relative risks and estimates of communality**

| **Risk factor** | **Relative risk^1^** | **Communality** |
| --- | --- | --- |
| Less education | 1.6 | 0.55 |
| Hypertension | 1.2 | 0.58 |
| Obesity | 1.3 | 0.44 |
| Hearing loss | 1.4 | 0.28 |
| Smoking | 1.3 | 0.36 |
| Depression | 2.2 | 0.43 |
| Physical inactivity | 1.2 | 0.62 |
| Social isolation | 1.6 | 0.43 |
| Diabetes | 1.7 | 0.36 |
| Air Pollution | 1.1 | 0.65 |
| Alcohol | 1.2 | 0.41 |
| Vision | 1.5 | 0.35 |
| High Cholesterol | 1.3 | 0.57 |

**Table s2: Variables included in the NZDep2018** **^2^**

Variable (proportions in small areas) in order of decreasing weight in the index

People with no access to the Internet at home

People aged 18 – 64 receiving a means tested benefit

People living in equivalised* households with income below an income threshold

People aged 18 – 64 unemployed

People aged 18 – 64 without any qualifications

People not living in own home

People aged <65 living in a single-parent family

People living in equivalised* households below a bedroom occupancy threshold

People living in dwellings that are always damp and/or always have mould greater than A4 size

* Equivalisation refers to “measures of the relative incomes needed by different types of families to attain the same material standard of living”

**Calculation of PAF**

The PAF is calculated using the formula

(PAF)= Pe(RRe – 1) / (1 + Pe[RRe – 1]) (1)

Where Pe is the prevalence of the exposure and RRe the relative risk of disease because of that exposure·

We then calculated overall PAF:

PAF=1 – [(1 – PAF1)(1 – PAF2)(1 – PAF3)...] (2)

Each individual risk factor’s PAF was weighted according to its communality using the formula:

Weight (w)=1 – communality

Weighting was included in the calculation of overall PAF using the formula: PAF=1 – [(1 – w*PAF1)(1 – w*PAF2)(1 – w*PAF3)...] (3)

**Calculation of communality**

We followed the methods described in the Lancet Commission^1^ using STATA

Step 1: Create a tetrachoric correlation matrix of 13 risk factors.

Step 2: Conduct principal components analysis of the matrix.

Step 3: Retain components with eigenvalues ≥1.

Step 4: Calculate communality as the sum of the square of all component loadings.

**Converting Hazard ratios to relative risk**

We converted calculated hazard ratios for neighbourhood deprivation to relative risk using the formula

RR = (1 - exp(-HR * ln(1-r))) / r^3^

where RR is the relative risk, HR the hazard ratio, and r the age specific incidence rate

Relative risks were then transformed into risk in the exposed (R_exp_) and unexposed (R_unexp_) following the methods outlined by Mukadam et al^4^ using the formulas

R_exp_ = RR /((p* RR_j_)+1-p) and R_unexp_ = 1/((p* RR)+1-p)

**Calculating the projected proportion of dementia prevented following a hypothetical intervention in midlife (40-64yrs) that reduces risk factor prevalence by 25%.**

Projections out to 30yrs modelled for the total NZ population, as well as for the four main ethnic groups

Figure S1: Markov model

Table S3. Base data required for the model and data availability by dementia stage, age band, and ethnicity.

|  | Dementia stage | Age band | Ethnicity |
| --- | --- | --- | --- |
| **Dementia** |  |  |  |
| Dementia incidence ^4,5^ | NA | Yes | No |
| Annual transition rates between dementia stages^4,5^ | Yes | NA | NA |
| **Mortality** |  |  |  |
| Excess mortality due to dementia ^4,5^ | Yes | NA | NA |
| Annual mortality rate ^6^ | NA | Yes | Yes |
| **Risk factor** |  |  |  |
| Deprivation | NA | Yes | Yes |
| Risk in exposed/unexposed | NA | Yes | Yes |

1. Incidence of dementia by 5yr age-band from age 40 years

Age specific incidence was derived from two sources:

Incidence for age 40-60yrs are from a 2023 metanalysis on the incidence of YOD by Hendriks et al. ^7^

Incidence for age 60+ uses NZ specific data ^8^

2. Annual Transition rates between dementia stages ^4,5^

We have used the same transition rates as those used by Mukadam et al. ^4^ (Table S3) These in turn have been derived from estimates by Spackman et al^9^ and Brookmeyer et al^10^ for the transition rates between dementia stages

Table S4: Transition matrix for Markov model

| Annual rates of transition between stages of dementia or death | | | | | |
| --- | --- | --- | --- | --- | --- |
|  | | to | | | |
|  |  | Mild | Moderate | Severe | Death |
| From | mild | 1-0.167-pop rate | O.167 | - | Pop rate |
|  | Moderate | - | 1-0.2- (pop rate + 0.11) | 0.2 | Pop rate + 0.11 |
|  | severe | - | - | 1-(pop rate + 0.11) | Pop rate + 0.11 |

Pop rate = population age-specific all-cause death rate

3. Annual Mortality rate by 5yr age-band from age 40 years and ethnicity

Annual mortality rates by age and ethnicity available from the Ministry of Health. ^6^ We have used mortality rates for 2021 as this is the latest data available.

4. Excess mortality due to dementia, by dementia stage

We have assumed no excess mortality risk in those with mild dementia. The excess mortality in those with moderate or severe dementia is estimated to be 11% per year.^11,12^

5. Deprivation prevalence by age-band and ethnicity from age 40 years

As detailed in the main article, the NZHS^13^ provides prevalence by age band and ethnicity for deprivation.

Supplementary Table S5: Total population attributable fraction (PAF) for 13 risk factors, by deprivation quintile and ethnicity

|  | Q1 | Q2 | Q3 | Q4 | Q5 |
| --- | --- | --- | --- | --- | --- |
| Maori | 33.3% | 40.4% | 42.3% | 44.4% | 47.1% |
| Pacific peoples | 30.9% | 42.8% | 37.9% | 43.5% | 44.2% |
| Asian | 27.5% | 29.6% | 29.8% | 33.7% | 39.9% |
| European | 32.8% | 35.9% | 38.0% | 42.1% | 45.5% |

*Deprivation Quintile: Q1 (least deprived) – Q5 (most deprived)*

Supplementary Table S6: Hazard ratios for change in deprivation levels

|  |  | HR | p | 95% CI |  |
| --- | --- | --- | --- | --- | --- |
| Quintiles | A | 1.23 | <.0001 | 1.11 | 1.36 |
|  | B | 1.21 | <.0001 | 1.11 | 1.33 |
|  | C | 1.29 | <.0001 | 1.17 | 1.43 |
|  | D | 1.37 | <.0001 | 1.19 | 1.57 |

A if the risk of the 20% most deprived people was reduced to that of the other 80%

B if the risk of the 40% most deprived people was reduced to that of the other 60%

C if the risk of the 60% most deprived people was reduced to that of the other 40%

D if the risk of the 80% most deprived people was reduced to that of the other 20%

Table s7: Modelled change in dementia prevalence projections, stratified by ethnicity, following hypothetical midlife interventions that shifts the dementia risk associated with deprivation under 4 progressively more radical scenarios: a) shifting risk of Q5 to that of Q1-4 b) shifting risk of Q4-5 to that of Q1-3 c) shifting risk of Q3-3 to that of Q1-2 or d) shifting the risk of Q2-5 to that of Q1

|  |  | Maori | Pacific | Asian | Euro |
| --- | --- | --- | --- | --- | --- |
| A | 10yrs | 2.2% | 2.8% | 0.8% | 0.5% |
|  | 20yrs | 4.8% | 6.4% | 1.8% | 1.4% |
|  | 30yrs | 6.9% | 9.4% | 3.0% | 2.5% |
| B | 10yrs | 3.3% | 3.5% | 1.6% | 1.0% |
|  | 20yrs | 7.1% | 8.2% | 3.6% | 3.0% |
|  | 30yrs | 10.4% | 12.3% | 6.0% | 5.5% |
| C | 10yrs | 5.2% | 5.3% | 3.1% | 2.0% |
|  | 20yrs | 11.4% | 12.7% | 6.9% | 6.1% |
|  | 30yrs | 17.2% | 19.4% | 12.0% | 11.6% |
| D | 10yrs | 6.5% | 6.8% | 4.3% | 3.1% |
|  | 20yrs | 14.8% | 16.5% | 10.0% | 9.5% |
|  | 30yrs | 22.7% | 25.9% | 17.9% | 18.8% |

A if the risk of the 20% most deprived people was reduced to that of the other 80%

B if the risk of the 40% most deprived people was reduced to that of the other 60%

C if the risk of the 60% most deprived people was reduced to that of the other 40%

D if the risk of the 80% most deprived people was reduced to that of the other 20%

REFERENCES:

1.	Livingston G, Huntley J, Liu KY, et al. Dementia prevention, intervention, and care: 2024 report of the Lancet standing Commission. *Lancet* 2024; **404**(10452): 572-628.

2.	Atkinson J SC, Crampton P. NZDep2018 Index of Deprivation, Interim Research Report. Wellington: University of Otago, 2019.

3.	VanderWeele TJ. Optimal approximate conversions of odds ratios and hazard ratios to risk ratios. *Biometrics* 2020; **76**(3): 746-52.

4.	Mukadam NA, R; Knapp, M; Wittenberg, R; Karagiannidou, M; Costafreda, SG; Tutton, M; Alessi, C; Livingston, G. Effective interventions for potentially modifiable risk factors for late-onset dementia: a costs and cost-effectiveness modelling study. *The Lancet Healthy Longevity* 2020; **1**(1): e13-e20.

5.	Norton S, Matthews FE, Barnes DE, Yaffe K, Brayne C. Potential for primary prevention of Alzheimer's disease: an analysis of population-based data. *Lancet Neurol* 2014; **13**(8): 788-94.

6.	Health Mo. Mortality web tool. <https://www.health.govt.nz/publication/mortality-web-tool>.

7.	Hendriks S, Peetoom K, Bakker C, et al. Global incidence of young-onset dementia: A systematic review and meta-analysis. *Alzheimers Dement* 2023; **19**(3): 831-43.

8.	Ma'u E, Cullum S, Mukadam N, Davis D, Rivera-Rodriguez C, Cheung G. Estimating the incidence of dementia in New Zealand: a cohort study applying capture-recapture modelling to routinely collected linked health datasets. *Lancet Reg Health West Pac* 2024; **52**: 101216.

9.	Spackman DE, Kadiyala S, Neumann PJ, Veenstra DL, Sullivan SD. Measuring Alzheimer disease progression with transition probabilities: estimates from NACC-UDS. *Curr Alzheimer Res* 2012; **9**(9): 1050-8.

10.	Brookmeyer R, Evans DA, Hebert L, et al. National estimates of the prevalence of Alzheimer's disease in the United States. *Alzheimers Dement* 2011; **7**(1): 61-73.

11.	Brookmeyer R, Johnson E, Ziegler-Graham K, Arrighi HM. Forecasting the global burden of Alzheimer's disease. *Alzheimers Dement* 2007; **3**(3): 186-91.

12.	Johnson E, Brookmeyer R, Ziegler-Graham K. Modeling the effect of Alzheimer's disease on mortality. *Int J Biostat* 2007; **3**(1): Article 13.

13.	Health Mo. New Zealand Health Survey: Annual data explorer. 2024. <https://minhealthnz.shinyapps.io/nz-health-survey-2023-24-annual-data-explorer/_w_5dacb90c3fd848f5902554edcc51ba33/#!/home> (accessed March 2024).
